# A clinical analysis of vitrectomy for severe vitreoretinopathy in patients with chronic renal

**DOI:** 10.1186/s12886-018-0704-7

**Published:** 2018-02-08

**Authors:** Long Su, Guannan Huang, Songtao Yin, Xia Hua, Xin Tang

**Affiliations:** 1 Department of Ophthalmology, The 2nd Hospital of Tianjin Medical University, Tianjin Institute of orbital Disease, Tianjin, China; 2 Tianjin Eye Hospital, Tianjin Key Laboratory of Ophthalmology and Vision Science Clinical College of Ophthalmology, Tianjin Medical University , No. 4 Gansu Rd, Heping District, Tianjin, 300020 China

**Keywords:** Chronic renal failure, Vitreoretinopathy, Vitrectomy

## Abstract

**Background:**

The recent advancement in the management of chronic renal failure (CRF) has significantly increased the longevity of the patients, which increase the incidence of severe vitreoretinopathy. The vitrectomy is highly risky in this particular group of patients due to their systemic comorbidity. The timing surgical intervention is usually delayed because of the systemic conditions. This study is to evaluate the safety and effectiveness of 25-guage vitrectomy for severe vitreoretinopathy in the CRF patients.

**Methods:**

In this retrospective study, 16 eyes of 16 CRF patients with severe vitreoretinopathy were undergone 25-guage vitrectomy in the department of Ophthalmology of the Second Hospital of Tianjin Medical University from February 2015 to April 2017. The visual outcome, complications and perioperative medical management were documented and analyzed.

**Results:**

The best-corrected visual acuity(BCVA)of fourteen eyes were lower than 20/200 preoperatively. Surgery duration ranged from 28 to 72 min, with a mean of 48.4 ± 13.6 min. During the surgery, 12 eyes were diagnosed with DR, while two them were complicated with tractional retinal detachment and one with branch retinal vein occlusion. Three eyes were diagnosed with branch retinal vein occlusion, and one eye was diagnosed with hypertensive retinopathy. Postoperative BCVA of six eyes ≥20/40, seven eyes ≥20/200, and three eyes < 20/200. BCVA of eight eyes improved more than three lines, three eyes improved two lines, and four eyes improved one line. BCVA decreased from hand movement to light perception in one patient who developed neovascular glaucoma two weeks after surgery.

**Conclusion:**

In chronic renal failure patients with severe vitreoretinopathy, the well-planned minimally invasive vitrectomy is effective and safe. Additionally, careful management of the perioperative systemic conditions is important to improve the visual acuity and quality of life as well.

## Background

Chronic renal failure (CRF) is a condition that results from the progression of various chronic kidney diseases. The major etiologies of CRF include diabetic nephropathy, hypertensive renal arteriolar sclerosis, glomerulonephritis, renal interstitial disease, renal vascular disease, and inherited renal disease. These conditions can severely affect the overall health of patients. However, the life expectancy and quality has been significantly improved since the application of peritoneal dialysis and hemodialysis. Therefore, patients that develop CRF are at an increased risk of developing secondary complications,such as uncontrolled diabetes mellitus (DM) and hypertension, which often lead to severe vitreoretinopathy. The complications of CRF, including hypoalbuminemia, anemia, toxin accumulation in the blood, and coagulation dysfunction are all risk factors exacerbating retinopathy [1]. Some studies have suggested that the incidence of hypertensive retinopathy is approximately 68% in CRF patients combined with hypertension [2], whereas the incidence of diabetic retinopathy (DR) is as high as 88.7% in CRF patients combined with DM [3].

CRF patients combined with severe vitreoretinopathy often have vitreous hemorrhage and tractional retinal detachment that can be treated by vitrectomy. Given the complicated comorbid conditions of these patients, the surgery is highly risky. For instance, the use of anticoagulation during hemodialysis would increase the risk of bleeding after surgery. Hypoproteinemia could lead to poor wound healing and the electrolyte disorders can lead to arrhythmia, heart failure and cardiac arrest. The resilient hypertension could increase the bleeding. The anemia could increase the possibility of the ischemic optic neuropathy [4]. Many patients lose the opportunity for surgical treatment because of these severe complications. In the absence of timely and effective therapy, many CRF patients eventually become blind. The recent advancement in minimal invasive vitrectomy effectively shortens the surgery duration and reduces the incidence of both intraoperative and postoperative complications. Additionally, the smaller incisions provide the opportunity of promote wound healing and visual recovery in those critical patients [5, 6]. We used 25-gauge vitrectomy to treat severe vitreoretinopathy in patients with CRF and retrospectively analyzed and reported the clinical outcomes as below.

## Methods

The Ethics Committee at Tianjin Medical University approved this study. All patients gave informed consents. Sixteen patients with stage 4 and 5 chronic kidney diseases according to the USA National Kidney Foundation’s staging system underwent vitrectomy in the department of Ophthalmology of the Second Hospital of Tianjin Medical University from February 2015 to April 2017. The inclusion criteria in this study were glomerular filtration rates of < 30 mL/min per 1.73 m^2^, and the course of disease was ≥3 months.

### General preoperative conditions

Thirteen male patients and three female patients aged are involved in this study. The mean age of the patients was 52.9 years (ranged from 32 to 63 years old). Among these patients, 1 of them was in the thirties, 1 of them was in the forties, 11 of them were in their fifties and 3 of them was in their sixties. The course of disease ranged from 4 months to 9 years, with a mean of 3.2 years. Ten patients were on hemodialysis, two patients underwent peritoneal dialysis, and four patients did not have any dialysis. Fourteen patients were combined with DM with an average course of 16.3 years (ranged from 1 to 25 years). All patients were combined with hypertension. The average course of hypertension was 7.5 years (ranged from 0.4 to 15 years, Table 1).

**Table 1:** General preoperative conditions of patients

No	HBp	DM	RD	Treatment	Hb	GHb	Scr	SK	echocardiography	EF
	(years)	(years)	(years)		(g/L)	(%)	(μmol/L)	(mmol/L)		(%)
1	10	20	2	HD	120	7.5	596.3	5.6	b,c,d,e,f	36
2	5	24	3	HD	87	6.8	712	5.3	a,b,d,e	52
3	0.3	20	1	Drug	128	8.7	422	4.8	a,b,c	58
4	13	15	2	HD	79	6.2	860	4.8	a,b,d,e	52
5	12	25	3	HD	117	7.6	720	4.2	a,b,e	51
6	7	12	9	PD	107	6.4	650	6.2	a,b,d,e	52
7	10	16	6	Drug	112	5.8	249	5.8	a,b,d,e	54
8	10	10	4	HD	89	5.4	1095	4.7	a,b,c,d	54
9	10	22	3	PD	102	6.5	771	4.1	a,b,d	50
10	1	13	1	HD	107	7.1	1081	5.3	a,b	68
11	1	13	1	HD	95	6.8	1029	5.1	a,b	68
12	1	1	1	HD	80	6.2	893	5.5	a,b,e	58
13	5	10	5	Drug	98	7.5	493	4.6	a,b,d	60
14	3	24	4	HD	128	9.5	691	5.6	a,b,e	60
15	10	0	0.3	HD	109	4.5	459	4.3	a,b,e	62
16	15	0	4	Drug	122	4.8	243	5.1	a,b	65

*HBp* hypertension, *DM* diabetic mellitus, *RD* renal disease, *Hb* hemoglobin, *GHb* glycolated hemoglobin, *HD* hemodialysis, *PD* peritoneal dialysis, *Scr* serum creatinine, *SK* Serum kalium, *EF* ejection fraction, *a* Left atrial enlargement, *b* Left ventricular dysfunction, *c* pulmonary arterial hypertension, *d* hydropericardium, *e* aortic valve calcification, *f* cardiomegaly

Twelve patients had decreased hemoglobin, five had elevated level of serum potassium, and five had low cardiac function. Sixteen eyes had vitreous hemorrhage, and two were complicated with tractional retinal detachment. All patients were subjected to the following ophthalmic examinations: best corrected visual acuity (BCVA), slit lamp microscopy, mydriatic fundus examination, gonioscopy, A-scan ultrasonography, B-scan ultrasonography, and specular microscopy. The patients also underwent a series of systemic evaluations, including full blood cell count, hepatic and renal function tests, electrolytes, blood glucose, glycosylated hemoglobin measurements, electrocardiography, and echocardiography.

### Surgery

Patients on hemodialysis underwent heparin-free hemodialysis treatment one day before the surgery and repeated within seven days after surgery. They received normal anticoagulant dialysis afterwards. Seven eyes received intravitreal injection of anti-vascular endothelial growth factor (anti-VEGF) 0.05 ml (Conbercept, KH902; Chengdu Kanghong Biotech Co., Ltd., Sichuan, China) three days before surgery.

During the operation, patients received retrobulbar injection of anesthesia. Fourteen patients showed an increase of blood pressure after the three scleral cannulas were inserted. They were treated with intravenous infusion of 1 mg midazolam for sedation and 12.5 mg urapidil for blood pressure. During the surgery, nine patients showed the increase of blood pressure, and four of them reached to a peak value of 210/110 mmHg. These patients were treated with an intravenous injection of 12.5 mg urapidil every 15 min, up to four times as needed or intravenous drip of 100 mg urapidil in 250 ml normal saline to maintain the blood pressure below 160/90 mmHg. Two patients showed a blood pressure consistently higher than 200/100 mmHg. The surgery was resumed and completed until the blood pressure was stabilized by intravenous trinitroglycerin.

The surgeries were performed by using Alcon Constellation 25-G vitrectomy machines (Alcon Laboratories, Inc., Fort Worth, TX, USA). After core vitrectomy, the cortical vitreous was stained with triamcinolone acetonide, and removed after the posterior vitreous detachment was created. The electrocoagulation was used for hemostasis. The proliferative epiretinal membrane was dissected to release the tangential traction. These were followed by pan retinal laser photocoagulation. Three eyes were filled with silicone oil due to the inferior retinal breaks. Patients combined with cataract underwent phacoemulsification and hydrophobic intraocular lens implantation. At the end of the surgery, 1 mg triamcinolone acetonide was injected into the vitreous cavity. Follow up examinations including visual acuity, intraocular pressure, slit lamp microscopy, and mydriatic fundus examination,fundus photography and macular OCT were conducted at 1 day,1 week, 4 weeks,and 12 weeks,postoperatively.

## Results

The best-corrected visual acuity (BCVA) before the operation were range from hand motion to 20/100. The mean surgery duration was 48.4 ± 13.6 min (ranged from 28 to 72 min). During the surgery, 12 eyes were diagnosed as DR, with two of them showing tractional retinal detachment and one complicated with branch retinal vein occlusion. Three eyes were diagnosed as branch retinal vein occlusion, and one eye was diagnosed as hypertensive retinopathy. Postoperative BCVA of 6 eyes ≥20/40, 7 eyes ≥20/200, and 3 eyes < 20/200. BCVA of 8 eyes improved more than 3 lines, 3 eyes improved 2 lines, and 4 eyes improved 1 line. BCVA decreased from hand movement to light perception in one patient who developed neovascular glaucoma two weeks after the surgery.

The surgical technique and intraoperative and postoperative conditions were described in Table 2. The most common postoperative complication was increased IOP, which occurred in 3 eyes. Two of them were controlled with topical medication. One eye developed neovascular glaucoma despite the treatment. Four eyes showed pre-retinal hemorrhage that resolved spontaneously after 2 weeks. Three eyes experienced vision fluctuation and retinal ischemia, both of which improved with pharmacological therapy within two weeks. One eye was diagnosed with endogenous fungal endophthalmitis after the placement of a hemodialysis venous catheter. With the intravitreal drug injection, the infection resolved 10 weeks after the surgery. One patient developed a respiratory tract infection and heart failure, which were controlled with internal medicine therapy two weeks after the surgery.

**Table 2:** The surgical methods and intraoperative and postoperative conditions

No	Etiology	BCVA pre-op	BCVA post-op	Treatment	Surgical	Surgery duration	BP enter	BPmax During	BP back to
		(Snellen)	(Snellen)	pre-OP	procedure	(minutes)	OR(mmHg)	OP(mmHg)	ward(mmHg)
1	PDR,TRD	HM	LP	/	PPV + P + I + PRP + SO	72	165/100	160/100	150/80
2	PDR,VH	FC	20/100	PRP	PPV + PRP	28	160/95	160/90	130/80
3	PDR,VH	FC	20/30	/	PPV + P + I + PRP	55	142/85	165/95	130/80
4	PDR,VH,ERM	20/200	20/40	PRP,IV Cbcp	PPV + P + I + PRP + IVTA	45	165/88	175/100	120/70
5	PDR,VH	20/100	20/50	PRP	PPV + P + I + PRP	52	150/90	160/100	150/80
6	PDR,VH	HM	20/500	IV Cbcp	PPV + P + I + PRP + IVTA	53	162/92	160/90	150/90
7	PDR,TRD	HM	20/200	IV Cbcp	PPV + P + I + PRP + SO	62	185/102	190/100	160/90
8	PDR,BRVO	HM	20/150	PRP,IV Cbcp	PPV + P + I + PRP + IVTA	45	180/100	180/90	130/80
9	PDR.VH	FC	20/100	PRP	PPV + P + I + PRP + IVTA	50	162/95	180/80	140/80
10	PDR,VH	FC	20/20	IV Cbcp	PPV + P + I + PRP + IVTA	48	150/92	160/90	150/90
11	PDR,VH	FC	20/25	/	PPV + P + I + PRP + IVTA	52	158/89	160/90	150/90
12	VH,BRVO	HM	20/60	/	PPV + P + I + LRP + IVTA	62	220/105	175/95	140/80
13	VH,BRVO	20/400	20/30	/	PPV + LRP + SO	62	138/85	160/80	160/75
14	PDR,VH,BRVO	20/500	20/40	IV Cbcp	PPV + P + I + PRP + IVTA	32	145/92	200/100	130/80
15	HR	20/400	20/100	/	PPV + PRP + IVTA	28	230/110	200/110	170/110
16	PDR,VH	20/500	20/300	IV Cbcp	PPV + PRP + IVTA	28	200/100	150/90	130/80

*BCVA* best corrected visual acuity, *pre-op* pre-operation, *post-op* post-operation, *BP* blood pressure, *OR* operating room, *OP* operation, *BPmax* blood pressure maximum, *PDR* proliferative diabetic retinopathy, *TRD* tractional retinal detachment, *VH* vitreous hemorrhage, *BRVO* branch retinal vein occulusion, *ERM* epiretinal membrane, *HR* Hypertensive retinopathy, *HM* hand move, *FC* finger count, *LP* light perception, *PRP* pan-retinal photocoagulation, *IV Cbcp* intravitreal conbercept, *PPV* pars plana vitrectomy, *P* phacoemulsification, *I* intraocular lenses implantation, *IVTA* intravitreal triamcinolone acetonide, *SO* silicon oil, *LRP* local retinal photocoagulation

## Discussion

### CRF and severe Vitreoretinopathy

CRF is a clinical syndrome presenting as metabolites retention, disturbances in water-electrolyte homeostasis and acid-base metabolism, and dysfunction of various organ systems in the body. It is a systemic problem involving many aspects of the patient. The complications of CRF, including DM, hypertension, and anemia, are important causes of vitreoretinopathy [4]. Additionally, the long-term administration of heparin anticoagulant drugs during hemodialysis also increases the risk of vitreous hemorrhage [7]. In more advanced cases, the eyes develop dense vitreous hemorrhage and tractional retinal detachment.

The current study suggests that by systemically considering the general and ophthalmic conditions, reducing intraoperative and postoperative incidence of systemic and ophthalmic complications, and decreasing the intraoperative and postoperative hemorrhage, ophthalmologists could perform surgeries to improve the visual function of the CRF patients with the support of a competitive medical team. Vitrectomy is an effective approach to treat severe vitreoretinopathy [8]. Prudent analysis of surgical indications, appropriate surgical techniques and timing, are critical for postoperative recovery of visual function. Patients with CRF are often combined with refractory hypertension, heart failure, and electrolyte disturbance, which often delay their ophthalmic surgeries. It is not prudent to perform vitrectomy without considering the comorbid conditions. However, overemphasizing the risks of surgery or even deciding against surgery, could increase the patient’s risk of becoming permanently blind. In this series of patients, 13 out of 16 were younger than 60. It is important to tackle their ocular pathologies and improve their visual function, which could improve their independence of daily activities and increase the quality of life. Previous study also showed that patients with diabetic nephropathy who underwent dialysis could safely undergo vitrectomy [9]. Therefore, with careful management of the systemic conditions, ophthalmologists could perform vitrectomy after proactively stabilizing the systemic conditions [10].

### Perioperative characteristics and treatments of CRF patients

CRF Patients often have multiple abnormal indexes in the body. Recognizing the possible risks of synergetic effects of various abnormal indexes under the surgical stress state is pivotal to perioperative management of patients with CRF. Before the operation, a comprehensive assessment of general conditions and correction of abnormal indexes can reduce the possible risk of damaging heart, brain, and kidney function during the perioperative period.

1. Anemia. Renal failure affects the formation of erythropoietin and thereby affects hematopoietic function. Hemodialysis leads to loss of erythrocytes to different extents. Additionally, patients on hemodialysis are usually combined with erythropenia and hemoglobin reduction. If anemia is not corrected, postoperative optic nerve and retinal ischemia may lead to severe visual impairment [11]. Therefore, recombinant human erythropoietin and iron sucrose can be administered preoperatively to increase the levels of erythrocytes and hemoglobin.
2. Hypertension. CRF Patients are usually combined with refractory hypertension, which increase the risk of intraoperative and postoperative hemorrhage resulting in poorer surgical outcome. All patients participated in this study had certain level of hypertension. Outside the perioperative period, the patients could be treated with a calcium antagonist and angiotensin receptor blocker to control the blood pressure, while in the perioperative period, especially in the intraoperative period, the patients should be treated with short-acting anti-hypertensive drugs, such as urapidil, which can be metabolized quickly. In our series, most of the patients showed an increased blood pressure during the operation. Some of them showed a repeated increase in blood pressure higher than the critical values. We treated the patients with sedative (1 mg midazolam) before the surgery. And the urapidil was used accordingly during the surgery. Special attention should be given to control the blood pressure by using an infusion pump to prevent the exacerbation of renal failure due to acute renal injury caused by insufficient renal blood flow [12].
3. Heart failure. In this study, one patient developed heart failure after the surgery due to respiratory tract infection. The symptoms slowly resolved within 2 weeks after medical treatment. Renal failure is usually complicated with cardiac dysfunction, presenting with the malfunction of the enlarged left ventricle and significant decrease of ejection fraction. Some cases of renal failure are complicated with mild pericardial effusion. These patients could be treated with intravenous injection of levocarnitine to prevent cardiac disfunction.
4. Abnormality of serum creatinine, blood glucose, and electrolytes. Renal failure primarily presents with an increase in creatinine. However, the level of creatinine does not have a significant impact on vitrectomy. In this study, the levels of serum creatinine in patients were not rigorously restricted. The patients in this study had different courses of DM. The blood glucose levels of the dialysis patients were well controlled with glycosylated hemoglobin levels below 7.5%. With regards to electrolytes, serum potassium showed the highest increase. Hyperkalemia can lead to arrhythmia resulting in cardiac arrest. Perioperative hyperkalemia should be corrected with hemodialysis or intravenous infusion of a combination of insulin and high glucose.
5. Use of anticoagulant drugs. Patients with renal failure are usually treated with heparin for anticoagulation during hemodialysis. Heparin-induced coagulation anomaly is one of the most important factors determining whether vitrectomy would be successful. Nafamostat mesylate or low-molecular-weight heparin has been recommended in lieu of heparin prior to vitrectomy [13].
6. Postoperative posture. Many patients with renal failure also undergo hemodialysis. Because dialysis is conducted every two days, patients cannot maintain the prone position after the vitrectomy. Therefore, great care must be taken in vitrectomy for these patients to prevent tractional or iatrogenic retinal breaks.
7. Renal failure may lead to optic neuropathy [14], which is attributed not only to humoral factors, but also to primary ocular diseases. In this study, the eyes with DR constituted 81.3% (13/16), and the others had retinal vein occlusion and hypertensive retinopathy. During surgery, the optic disc was pale in 9 eyes, constituting 56.3% (9/16). Ten eyes had an arteriovenous ratio less than 1:2, constituting 62.5% (10/16). This was likely associated with optic nerve and retinal ischemia due to the arteriosclerosis and long-term hypertension. Because of the underlying vascular diseases, the possibility of these patients developing ischemic optic neuropathy is much higher than that of the other populations. The intraoperative intraocular infusion pressure and the change of postoperative intraocular pressure are more likely to result in ischemic optic neuropathy and retinopathy. During surgery, a normal intraocular infusion pressure should be maintained to prevent high intraocular pressure throughout the surgical process. Postoperative monitoring of intraocular pressure should be conducted closely. Upon the detection of any signs of ischemia, the patients should be treated with vasodilators to improve circulation and with trophic nerve drugs.
8. A poor systemic immune status. One patient in this study developed hyperpyrexia, septicemia, and endogenous fungal endophthalmitis after dialysis catheter placement at the 10th postoperative week. We considered that the local and systemic infection developed because of long-term dialysis, anemia, and low immunity observed in patients with CRF.

### Use of anti-VEGF drugs before vitrectomy

Patients with renal dysfunction typically have unfavorable systemic conditions., Therefore, the surgery duration should be shortened to ensure the safety of patients. As for patients with severe vitreoretinopathy, they should be first treated with an intravitreal injection of anti-VEGF drugs, and then treated with vitrectomy, so that the surgery duration can be effectively shortened. The anti-VEGF injection 3 to 7 days before the operation can promote recession of neovascularization and decrease the rate of intraoperative hemorrhage. This in turn will improve the visibility during the surgery, reduce the likelihood of iatrogenic retinal hole formation during membrane removal, and prevent unnecessary repetitive use of intraocular instruments [15]. The aforementioned factors reduce the change of postoperative complications [16–19]. Studies have shown that intraocular injection of anti-VEGF drugs will lead to recession of neovascularization. However, it may exacerbate the ischemia. Thus, it is not suitable for patients with severe ischemia. In addition, proliferative membranes without neovascularization would contract, which may exacerbate retinal detachment and result in traction-induced retinal breaks [20, 21]. Therefore, we performed intravitreal injection of anti-VEGF drugs on seven patients with severe vitreoretinopathy and conducted vitreoretinal surgery on the third day after injection, with no severe ischemia or hemorrhagic change during the surgery. All of them demonstrated a good therapeutic effect.

## Conclusion

In summary, although patients with CRF with systemic complications could affect postopererative outcomes, vitreoretinal surgery is not absolutely contraindicated in these patients. With the close collaboration with internal medicine team, careful perioperative management and relatively stable systemic conditions, a well-designed minimally invasive vitrectomy could be safe and effective to treat the vitreoretinopathy in patients with CRF to improve their visual acuity and quality of life.

## Abbreviations

BCVA: Best corrected visual acuity
BP: Blood pressure
BRVO: Branch retinal vein occlusion
CRF: Chronic renal failure
DM: Diabetes mellitus
DR: Diabetic retinopathy
EF: Ejection fraction
ERM: Epiretinal membrane
FC: Finger count
GHb: Glycolated hemoglobin
Hb: Hemoglobin
HBp: Hypertension
HD: Hemodialysis
HM: Hand move
HR: Hypertensive retinopathy
IVTA: Intravitreal triamcinolone acetonide
LP: Light perception
LRP: Local retinal photocoagulation
OR: Operating room
PD: Peritoneal dialysis
PDR: Proliferative diabetic retinopathy
PPV: Pars plana vitrectomy
PRP: Pan-retinal photocoagulation
RD: Renal disease
Scr: Serum creatinine
SK: Serum kalium
SO: Silicon oil
TRD: Tractional retinal detachment
VEGF: Anti- vascular endothelial growth factor
VH: Vitreous hemorrhage

## References

[1] Davis MD, Fisher MR, Gangnon RE, Barton F, Aiello LM, Chew EY, Ferris FL, Knatterud GL (1998). Risk factors for high-risk proliferative diabetic retinopathy and severe visual loss: early treatment diabetic retinopathy study report #18. Invest Ophthalmol Vis Sci.

[2] Vrabec R, Vatavuk Z, Pavlović D, Sesar A, Cala S, Mandić K, Bućan K (2005). Ocular findings in patients with chronic renal failure undergoing haemodialysis. Coll Antropol.

[3] Bajracharya L, Shah DN, Raut KB, Koirala S (2008). Ocular evaluation in patients with chronic renal failure--a hospital based study. Nepal Med Coll J.

[4] Mullaem G, Mitchell H (2012). Rosner ocular problems in the patient with end-stage renal disease. Semin Dial.

[5] Yokota R, Inoue M, Itoh Y, Rii T, Hirota K, Hirakata A (2015). Comparison of microinsicion vitrectomy and conventional 20-gauge vitrectomy for severe proliferative diabetic retinopathy. Jpn J Ophthalmol.

[6] Schrader WF, Josifova T (2010). The options to minimize the surgical trauma to treat ocular diabetic complications and to improve postoperative recovery and quality of life require an individualized approach. EPMA J.

[7] Wang TJ, Wu CK, Hu CC, Keller JJ, Lin HC (2012). Increased risk of co-morbid eye disease in patients with chronic renal failure: a population-based study. Ophthalmic Epidemiol.

[8] Iwahashi-Shima C, Sato T, Bando H, Ikeda T, Emi K (2013). Anatomic and functional outcomes of 25-gauge vitrectomy for repair of eyes with rhegmatogenous retinal detachment complicated by proliferative vitreoretinopathy. Clin Ophthalmol.

[9] Hayashi H, Kurata Y, Imanaga Y, Goya K, Oshima K (1998). Vitrectomy for diabetic retinopathy in patients undergoing hemodialysis for associated end-stage renal failure. Retina.

[10] Yan H, Yu JG, Han JD (2011). Efficacy of perioperative management for vitrectomy of patients with severe systemic disease. Chin J Ocul Fundus Dis.

[11] Hejsek L, Dusová J, Stepanov A, Rozsíval P (2013). Vision loss after uncomplicated pars plana vitrectomy. Cesk Slov Oftalmol.

[12] Meersch M, Schmidt C, Zarbock A (2016). Patient with chronic renal failure undergoing surgery. Curr Opin Anaesthesiol.

[13] Nakao T, Inaba M (2015). Masanori. Best practice for diabetic patients on hemodialysis 2012. Ther Apher Dial.

[14] Winkelmayer WC, Eigner M, Berger O, Grisold W, Leithner C (2001). Optic neuropathy in uremia: an interdisciplinary emergency. Am J Kidney Dis.

[15] Zaman Y, Rehman AU, Memon AF (2013). Intravitreal Avastin as an adjunct in patients with proliferative diabetic retinopathy undergoing pars plana vitrectomy. Pak J Med Sci.

[16] Shi L, Huang Y-F (2012). Postvitrectomy diabetic vitreous hemorrhage in proliferative diabetic retinopathy. J Res Med Sci.

[17] Blum A, Socea D, Ben-Shushan RS, Keinan-Boker L, Naftali M, Segol G, Tamir S. A decrease in VEGF and inflammatory markers is associated with diabetic proliferative retinopathy. Eur Cytokine Netw. 2012; 10.1684/ecn.2012.0321. 10.1684/ecn.2012.0321 23328414

[18] Morera Y, González R, Lamdan H, Pérez L, González Y, Agüero J, Castro J, Romero JC, Etchegoyen AY, Ayala M, Gavilondo JV (2014). Vaccination with a mutated variant of human vascular endothelial growth factor(VEGF) blocks VEGF—induced retinal neovascularization in a rabbit experimental model. Exp Eye Res.

[19] Su L, Ren X, Wei H, Zhao L, Zhang X, Liu J, Su C, Tan L, Li X (2016). Intravitreal Conbercept (KH902) for surgical treatment of severe proliferative diabetic ertinopathy. Retina.

[20] Arevalo JF, Maia M, Flynn HW, Saravia M, Avery RL, Wu L, Eid Farah M, Pieramici DJ, Berrocal MH, Sanchez JG (2008). Tractional retinal detachment following intravitreal bevacizumab (Avastin) in patients with severe proliferative diabetic retinopathy. Br J Ophthalmol.

[21] Kumar A, Sehra SV, Thirumalesh MB, Gogia V (2012). Secondary rhegmatogenous retinal detachment following intravitreal bevacizumab in patients with vitreous hemorrhage or tractional retinal detachment secondary to Eales’ disease. Graefes Arch Clin Exp Ophthalmol.

